# Quantitative phosphoproteomic analysis identifies novel functional pathways of tumor suppressor *DLC1* in estrogen receptor positive breast cancer

**DOI:** 10.1371/journal.pone.0204658

**Published:** 2018-10-02

**Authors:** Yesim Gökmen-Polar, Jason D. True, Edyta Vieth, Yuan Gu, Xiaoping Gu, Guihong D. Qi, Amber L. Mosley, Sunil S. Badve

**Affiliations:** 1 Department of Pathology and Laboratory Medicine, Indiana University School of Medicine, Indianapolis, IN, United States of America; 2 Department of Biochemistry and Molecular Biology, Indiana University School of Medicine, Indianapolis, IN, United States of America; 3 Department of Medicine, Indiana University School of Medicine, Indianapolis, IN, United States of America; 4 Indiana University Melvin and Bren Simon Cancer Center, Indianapolis, IN, United States of America; University of South Alabama Mitchell Cancer Institute, UNITED STATES

## Abstract

Deleted in Liver Cancer-1 (*DLC1*), a member of the RhoGAP family of proteins, functions as a tumor suppressor in several cancers including breast cancer. However, its clinical relevance is unclear in breast cancer. In this study, expression of *DLC1* was correlated with prognosis using publicly available breast cancer gene expression datasets and quantitative Reverse Transcription PCR in cohorts of Estrogen Receptor-positive (ER+) breast cancer. Low expression of *DLC1* correlates with poor prognosis in patients with ER+ breast cancer with further decrease in metastatic lesions. The Cancer Genome Atlas (TCGA) data showed that down regulation of *DLC1* is not due to methylation or mutations. To seek further insights in understanding the role of *DLC1* in ER+ breast cancer, we stably overexpressed *DLC1*-full-length (*DLC1*-FL) in T-47D breast cancer cells; this inhibited cell colony formation significantly *in vitro* compared to its control counterpart. Label-free global proteomic and TiO_2_ phosphopeptide enrichment assays (ProteomeXchange identifier PXD008220) showed that 205 and 122 phosphopeptides were unique to *DLC1*-FL cells and T-47D-control cells, respectively, whereas 6,726 were quantified by phosphoproteomics analysis in both conditions. The top three significant clusters of differentially phosphopeptides identified by DAVID pathway analysis represent cell-cell adhesion, mRNA processing and splicing, and transcription regulation. Phosphoproteomics analysis documented an inverse relation between DLC1 expression and several phosphopeptides including epithelial cell transforming sequence 2 (ECT2). Decreased phosphorylation of *ECT2* at the residue T359, critical for its active conformational change, was validated by western blot. In addition, the ECT2 T359-containing phosphopeptide was detected in both basal and luminal patient-derived breast cancers breast cancer phosphoproteomics data on the Clinical Proteomic Tumor Analysis Consortium (CPTAC) Assay portal. Together, for the first time, this implicates ECT2 phosphorylation in breast cancer, which has been proposed as a therapeutic target in lung cancer. In conclusion, this data suggests that low expression of *DLC1* is associated with poor prognosis. Targeting ECT2 phosphopeptides could provide a promising mechanism for controlling poor prognosis seen in DLC1_low_ ER+ breast cancer.

## Introduction

Deleted in Liver Cancer 1 (*DLC1)*, encoding a Rho GTPase-activating protein (RhoGAP), was originally discovered as a gene deleted or downregulated in primary hepatocellular carcinomas (HCC) [1]. Further studies reported the downregulation of *DLC1* in other human cancers including lung, breast, renal, cutaneous melanomas, nasopharyngeal (NPC), esophageal, cervical, and prostate cancers [2–7]. *DLC1* has been shown to act as a tumor suppressor in a number of experimental cancer models [8–15]. Recent studies suggest the emerging role of *DLC1* as a metastasis suppressor gene in a number of cancers including breast cancer [8]. Restoration of *DLC1* in metastatic breast cancer cells (M4A4), to levels similar to its non-metastatic clone (NM2C5), inhibited cell migration and invasion *in vitro* and reduced pulmonary metastases in athymic mice [16]. Other studies also reported that *DLC1* suppresses breast cancer metastasis to the bone and lung [17, 18]. Overexpression of *GATA3*, associated with upregulation of *DLC1*, significantly decreased the pulmonary metastasis in nude mice [17]. Knockdown of *DLC1* enhanced bone colonization of circulating cancer cells, whereas its restoration significantly decreased bone metastasis [18].

The clinical relevance of *DLC1* and its potential utility have not been well established in breast cancer. Here, we report the prognostic and mechanistic relevance of *DLC1* restoration in ER+ breast cancer.

## Materials and methods

### Analysis of publicly available datasets

Expression of *DLC1* was analyzed based on ER-status, molecular subtypes, and other clinico-pathological parameters using the datasets from the gene expression-based outcome for breast cancer online algorithm (GOBO)[19]. GOBO is a web-based analysis tool that utilizes 11 publicly available Affymetrix U133A gene expression data curated from 1881 breast cancer patients with associated stage, grade, nodal status, and intrinsic molecular classification [19]. The distribution of 1881 tumors was as follows: 1) ER positive tumors (n = 1225), 2) ER-negative tumors (n = 395), 3) systemically untreated patients (n = 927), and 4) patients treated with tamoxifen alone (n = 326). Clinical characteristics of individual datasets have been described previously [19]. Association of outcome was investigated for each patient cohort with overall survival (OS) as endpoint and 10-year censoring in the above groups. The Kaplan-Meier survival analysis was calculated using Cox proportional hazard model, and the score test of the proportional hazard model is equivalent to the log-rank test.

Somatic mutation rate, DNA copy number alterations (CNAs), mRNA, and methylation status for *DLC1* were analyzed using the cBioPortal for Cancer Genomics (http://cbioportal.org). The portal is a Web resource to analyze complex cancer genomics data including genetic, epigenetic, gene expression and proteomic events [20, 21]. All tumor samples that have CNA and sequencing data (n = 963 samples) and methylation data (HM450; n = 784 patients) were analyzed.

### Patient samples

All protocols were reviewed and approved by the Institutional Review Board of Indiana University. Samples and clinical records were anonymized prior to access by the authors and linked with a numerical identifier. The IRB waived requirement of informed consent. All archival formalin-fixed, paraffin-embedded (FFPE) tumor blocks in this study were from patients with ER-positive (greater than 1% expression as per ASCO-CAP guidelines) breast carcinomas at the Indiana University Health Pathology Lab (IUHPL). The quality of each block and the relative cellular composition were determined by the histopathological assessment of adjacent sections from FFPE tumor blocks.

### Onco*type* DX samples

Sixty archival FFPE tumor blocks were obtained from patients with ER-positive and node-negative breast carcinomas at IUHPL based on their Onco*type* DX recurrence score (19 low score, 21 intermediate score, and 20 high score). Four of the 60 cases had a lobular histology. Demographic and clinical characteristics of the patients were acquired from medical charts as described previously [22].

### Primary ER-positive breast tumors and matched lymph node samples

Twenty-three archival FFPE primary human breast tumor blocks and their matched lymph node metastases were obtained from patients with ER-positive breast carcinomas at the IUHPL (**S1 Table)**.

### RNA isolation and quantitative reverse transcription-PCR (qRT-PCR)

Total RNA was extracted from 10μm-thick sections of archival FFPE blocks using RecoverAll total nucleic acid isolation kit (Life Technologies, Grand Island, NY). The quality of RNA was assessed using the Nanodrop ND-1000 spectrophotometer (Thermo Fisher Scientific, Waltham, MA). Total RNA from breast cancer cell lines was isolated using miRNeasy kit (Qiagen, Germantown, MD). Total RNA was reverse-transcribed using the high capacity cDNA reverse transcription kit (Thermo Fisher Scientific, Waltham, MA) according to the manufacturer’s instructions. The mRNA level of *DLC1* (Hs00183436_m1) and *ECT2* (Hs00978168_m1) was analyzed by quantitative reverse transcription- PCR (qRT-PCR) using TaqMan gene expression assays on an ABI Prism 7900 platform (Thermo Fisher Scientific, Waltham, MA) with *ACTB* (Hs00357333_g1) and *GUSB* (Hs99999908_m1) as endogenous controls for normalization. All qRT-PCR reactions were performed in duplicates for tumor blocks and triplicates for breast cancer cell lines. The gene expression values was analyzed according to ∆∆Ct method using the Applied Biosystems DataAssist Software v3.0. GraphPad Prism 7.03 software was used to analyze statistical significance for qRT-PCR data.

### Breast cancer cell lines and generation of stable full-length DLC1 (DLC1-FL) construct

MCF-7, T-47D and ZR75.1 cell lines were purchased from American Type Culture Collection (ATCC, Manassas, VA). Cell lines have been carefully maintained in a humidified tissue culture incubator at 37°C in 5% CO_2_:95% air atmosphere, and stocks of the earliest passage cells have been stored. The cell lines were grown in phenol-red–free DMEM containing 5% charcoal-stripped fetal calf serum (CCS) and 100 mg/mL penicillin at least 4 days before the experiments as described previously [23].

To confirm the functional relevance of *DLC1* in ER-positive breast cancer, we overexpressed full-length *DLC1* (*DLC1-FL*) in T-47D breast cancer cell line using GenScript's GenEZ ORF *DLC1* construct *(DLC1-FL; NM_182643)*. GenScript's GenEZ ORF *DLC1-FL* was cloned into mammalian expression cloning vector, pcDNA3.1+/C-(K)-DYK, using the CloneEZ cloning technology, according to the manufacturer’s instructions (GenScript, Piscataway, NJ). Stable cells with the control vector (pcDNA3.1+/C-(K)-DYK vector only) or vector with DLC1-FL construct were generated in T-47D cells, which lacks *DLC1* expression. The colonies were screened using qRT-PCR (**S1 Appendix**) and the construct with high *DLC1* expression was chosen for further analysis. Protein expression was also validated using Western blot assays. Throughout the paper, T-47D cells with control vector and DLC1 overexpression are named as control and *DLC1-FL*, respectively.

### Clonogenic assay

Control and *DLC1-FL* cells (200 cells) were plated in triplicates in 6 well plates for 14 days to assess the cell growth. The media was changed every 3 days. After 14 days, media were aspirated and cells were stained with crystal violet. Cells were permeabilized using citrate buffer and absorbance was read at 560 nm on a plate reader.

### Cell-cycle analysis

Control and DLC1-FL cells (5x10^5^ cells/ml) were plated as described above (breast cancer cell lines) and harvested at 24 and 48 hours after the attachment of cells, suspended in PBS, and fixed in 70% ethanol. Then, DNA content was evaluated after propidium iodide staining. Fluorescence-activated cell-sorting analysis was carried out using a FACScan flow cytometer (Beckton Dickinson) and CellQuest software.

### Apoptosis assay

The PE Annexin V Apoptosis Detection Kit I (BD Biosciences) was performed by flow cytometry according to the manufacturer’s instructions. Briefly, *DLC1-FL* and control cells (5x10^5^ cells/ml) were harvested, washed in PBS, and pelleted by centrifugation. Cells were suspended with 1X binding buffer and then 5 μl Annexin V-fluorescein isothiocyanate was added. After 15 min, 10 μl propidium iodide was added and the suspension was incubated in the dark at room temperature for 15 min. Binding buffer (1X; 400 μl) was added to the suspension and was gently vortexed. Cells were analyzed using a FACScan flow cytometer. The numbers show the percentages of cells in each quadrant (bottom left: intact cells; bottom right: early apoptotic cells; top left: necrotic cells; top right: late apoptotic or necrotic cells).

### Invasion assay

The cell invasion assay was performed using CytoSelect 24 well cell invasion assay. Briefly, cells were plated in phenol red-free MEM with 5% charcoal stripped fetal calf serum for 48 hours and switched to 1% serum media for 24 hours. Cells were added to the upper chamber (6000 cells/insert) in serum-free medium. Cell were then allowed to migrate towards the lower chamber in the presence or absence of Fetal Bovine Serum (20%). After 72 hours, cells on the underside of the upper chambers were stained using the Cell Invasion Assay Kit (Promega, Madison, WI, USA) and examined by light microscopy according to the manufacturer’s instructions.

### Rho GTPase activation assay

RhoA activity was assessed with the Rhotekin binding assay as described previously (Cytoskeleton, Inc., Denver, CO). Briefly, cells were grown in 100-mm dishes. After serum starvation for 24 hours, cells were treated either with calpeptin (Rho Activator I; 0.1 mg/ml final) for 30 minutes or with carrier only (DMSO). Cell lysates were incubated with the GST-fused Rho-binding domain of Rhotekin immobilized on reduced glutathione-agarose for 2 hours at 4°C in a tube rotator and then rinsed. The level of active RhoA and total RhoA was detected by Western blotting with anti-RhoA antibody.

### Label-free quantitative global proteomic and phosphoproteomic analyses

Global proteomic and phosphoproteomic analyses of control and DLC1-FL cells were performed at the Indiana University School of Medicine Proteomics Core facility including cell extract preparation, protein digestion with LysC/trypsin, enrichment of phosphopeptides, and analysis via liquid chromatography (LC) coupled with tandem mass spectrometry (MS/MS) (**S1 Fig**). Cell pellets were lysed and proteins extracted with 8M urea in Tris-HCl pH 8.0 by sonication and the resulting supernatants were recovered by centrifugation. The resulting peptides were subjected to reduction with 5mM tris(2-carboxyethyl)phosphine hydrochloride (TCEP) and alkylated with 10mM chloroacetamide (CAM) prior to digestion with LysC (Roche) and Trypsin Gold (Promega), as previously described [24–26]. Peptide digestion reactions were cleaned up using a Waters Sep-Pak C18 column. Approximate 5% of the total peptide digestion was aliquoted for global proteomic analysis corresponding to ~50 μg of total protein. For phosphopeptide analysis, 1,000 μg of protein was phosphoenriched using a TiO_2_ column (Pierce Phosphoprotein Enrichment kit, Thermo Fisher, Waltham, MA) as per manufacturer’s instructions, and were cleaned up on a graphite column [27]. Briefly, ~20–25 μg digested total peptides or enriched phosphopeptides were injected onto a C18 Easy Spray 50 cm column and eluted with a 3 hour acetonitrile gradient (0–38%) in-line with a Thermo Orbitrap Velos Pro mass spectrometer [28]. MS1 data was acquired at a resolution of 60,000 with the top fifteen most intense ions selected for MS/MS analysis in the ion trap. Dynamic exclusion was applied for a 50 second duration. The resulting RAW files were analyzed in Thermo Proteome Discoverer (PD) 2.2. Database searches were performed with SEQUEST HT (as a node in PD 2.2) as previously described [26] with a few modifications: trypsin digestion, 2 maximum missed cleavages, precursor mass tolerance of 10 ppm, fragment mass tolerance of 0.8 Da, a fixed modification of +57.021 Da (carbamidomethylation) on cysteine, and variable modifications of +15.995 Da (oxidation) on methionine and +79.966 Da (phosphorylation) on serine, threonine, and tyrosine. A reverse database search was performed using SEQUEST HT to determine the spectral false discovery rate (FDR), and subsequent results were filtered by an FDR of ≤ 1% as previously described [24]. The FASTA database used was a human proteome downloaded from Uniprot on January 20, 2017 with addition of 73 common contaminants such as proteases and keratin, yielding a total of 21,010 non-redundant protein sequences. Prior to quantitation, the Spectrum Files RC node in PD 2.2 was utilized to perform spectrum recalibration. In addition, the MS1 intensity quantitative tool Minora Feature Detector was utilized for label-free quantitation for area under the curve calculations for up to the top three unique plus razor peptides detected per unique protein. Normalization was performed using the total peptide amount to account for random errors. Manual validation of spectra for phosphopeptides of interest was performed. The mass spectrometry proteomics data have been deposited to the ProteomeXchange Consortium via the PRIDE [29–31] partner repository with the dataset identifier PXD008220 and 10.6019/PXD008220.

Three biological replicates of control and DLC-FL cells were analyzed by mass spectrometry for both the global proteomic and the phosphoproteomic analyses in order to provide statistical rigor. Statistical significance was determined for the global proteome and phosphopeptide samples during PD 2.2 analysis using an ANOVA (background-based) followed by a Tukey HSD posthoc test (non-parametric test, which does not assume a normal distribution), and a *p*-value less than 0.05 was considered significant. An FDR-corrected *p*-value < 0.1 was also determined using the Benjamini-Hochberg correction. In addition, GO term analysis was performed in DAVID using the Uniprot accession numbers of the parent protein for the significantly changed phosphopeptides.

### Western blot analysis

Breast cancer cell lines were lysed in radioimmunoassay buffer (RIPA) and equal amounts of protein were subjected to SDS-PAGE and Western blot analysis in triplicates as described previously [32]. The Bio-Rad DC-Protein assay kit (Bio-Rad, Hercules, CA) was used to determine protein concentrations. Blots were incubated with antibodies against DLC1 (Thermo fisher Scientific, Waltham, MA), ECT2 (EMD Millipore, Burlington, MA), phospho-ECT2 [kind gift of Dr. Alan Fields [33]]. Antibodies against TFF1/pS2, cyclin D1, c-Myc, estrogen receptor α (ERα) and phospho-ERα (Ser118) were purchased from Cell Signaling Technology (Danvers, MA). Phospho-specific ECT2 antibody production and validation were described as previously [33]. Briefly, immunogenic phosphopeptides were: Ac-321CYLYEKANpTPELKKSV335-amide and Ac-321YLYEKANpTPELKKSVC335-amide respectively. Antibodies against GAPDH (GeneTex, Inc., Irvine, CA) and β-actin Sigma (St. Louis, MO) were used as loading controls. Protein bands were visualized by SuperSignal West Pico PLUS Chemiluminescent Substrate kit (Amersham, Piscataway, NJ) and Amersham Imager 600 GE Healthcare Life Sciences (GE Healthcare Bio-Sciences, Pittsburgh, PA). The data are representative of three individual sets.

### The Clinical Proteomic Tumor Analysis Consortium (CPTAC)

Published supplementary data from The Clinical Proteomic Tumor Analysis Consortium (CPTAC) breast cancer phosphoproteomics database [34] was downloaded from the Molecular and Cellular Proteomics website. Unique phosphopeptide sequences detected in the basal or luminal breast cancer analyses (performed on a Thermo Q Exactive) were compared to the sequences detected in this study (performed on a Thermo Velos Pro Orbitrap) to determine the overlap.

## Results

### Low expression of *DLC1* correlates with poor prognosis in patients with ER-positive breast cancer

**Fig 1:**
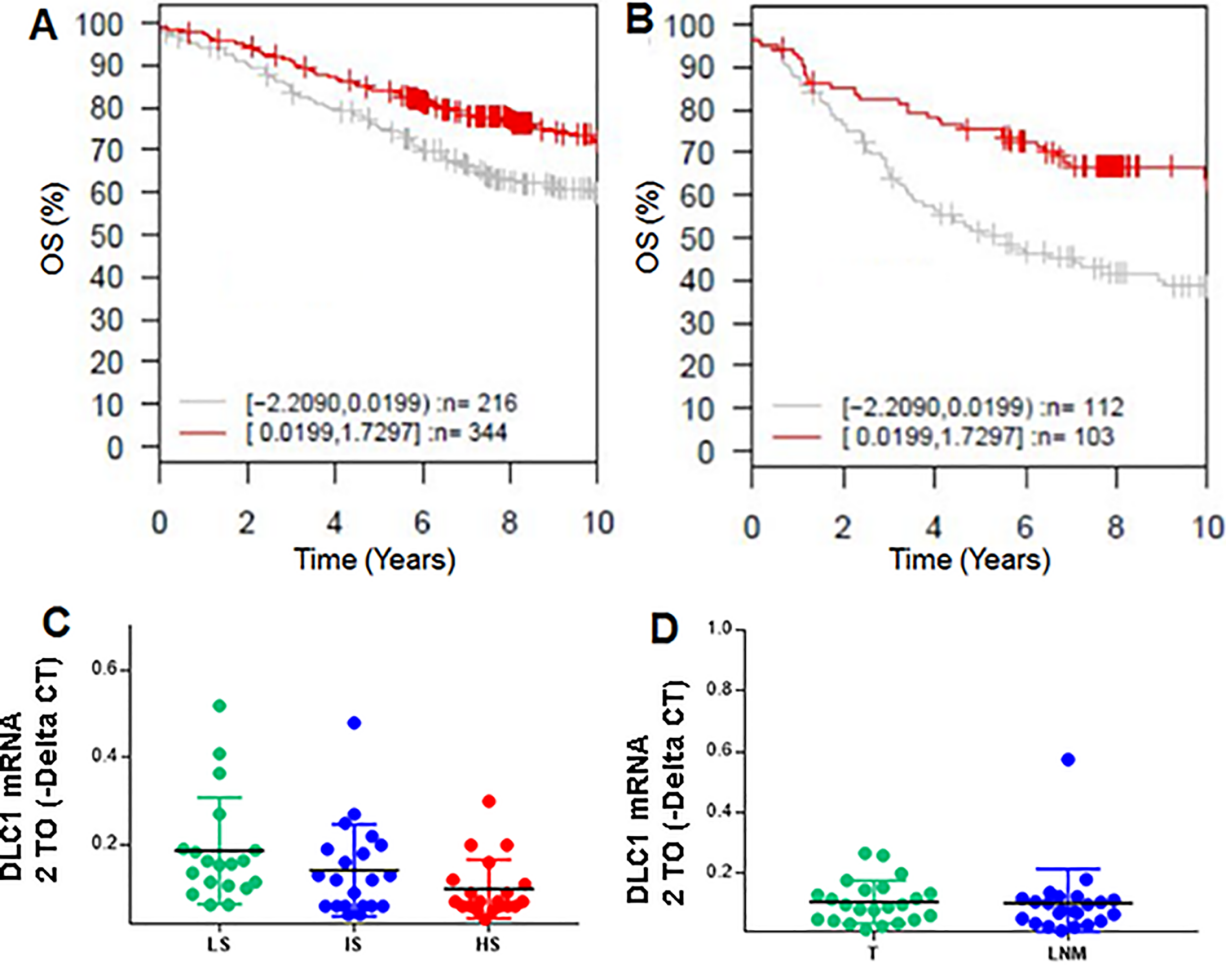
Low DLC1 expression is associated with poor prognosis in ER-positive breast cancer. Kaplan-Meier plots using GOBO online analysis (Affymetrix datasets) as endpoint for 10-year overall survival (OS) in **A**. ER-positive tumors (*p* = 0.002), and **B**. ER-positive and lymph node (LN)-positive tumors (*p* = 5e-04). High *DLC1* expression was > median; low expression (light grey), and high expression (red) expression. **C**. qRT-PCR assay showing that *DLC1* expression is lower in patients with High Recurrence Scores in an Oncotype DX cohort n = 60; LS-Low Score, IS-Intermediate Score and HS-High Score. *Adjusted *p* = 0.0160; statistically significant. **D.** qRT-PCR analysis showing independent set of lymph node metastatic samples (LNM), and their matched primary tumors (T)(n = 23 matched pairs; *p* = 0.2474). The expression values were analyzed according to ∆∆Ct method using the Applied Biosystems DataAssist Software v3.0. All FFPE cases were assayed in duplicates.

To determine the clinical relevance and prognostic value of *DLC1*, we first performed *in silico* analysis using Gene expression based Outcome for Breast cancer Online (GOBO) tool, which contains clinico-pathological and Affymetrix gene expression data from 1,881 breast cancer patients [19]. Of the 560 patients with ER-positive tumors and overall survival data, low expression of *DLC1* (40%) had significantly worse overall survival (OS) (ANOVA; *p* = 0.002; **Fig 1A**); an association was not observed in ER-negative tumors (ANOVA; *p* = 0.68; *data not shown)*. Expression levels of *DLC1* is especially is critical in patients with lymph node positive (LNpos) subset (ANOVA; *p* = 5e-04; **Fig 1B**), showing that cases with low *DLC1* (n = 52% of 215 patients with available data) have a significantly poor survival. This indicates that *DLC1* is prognostic in ER-positive patients and increasing the expression of *DLC1* could help to prevent recurrence/metastases.

We next sought to confirm this *in silico* data in a cohort of patient samples from Indiana University with Onco*type* DX recurrence score as a surrogate marker. This score has been validated in multiple clinical trials and is used in clinical practice to make treatment decisions [35, 36]. Analysis of the National Surgical Adjuvant Breast and Bowel Project (B14 and B20) clinical trials has led to the development of the Onco*type* DX recurrence score [35]. The score estimates the likelihood of disease recurrence in women with early-stage, ER-positive breast cancer and has been used as a surrogate for predicting outcomes. qRT-PCR analysis of 60 FFPE samples from a cohort of ER-positive, node-negative breast carcinomas with low, intermediate, and high (19, 21, and 20 cases, respectively) Onco*type* DX recurrence scores confirmed that patients with high risk of recurrence have lower levels of *DLC1* mRNA compared to low risk cases (Adjusted *p* = 0.016 using one-way ANOVA-multiple comparisons-GraphPad Prism 7.03) (**Fig 1C**). Expression of *DLC1* remained non-significant in cases with intermediate score compared to cases with low scores. These results suggest that low expression of *DLC1* indicates the likelihood of high recurrence and might contribute to innate resistance in ER-positive cancers.

The spread of breast tumors to local and regional lymph nodes is an important means of tumor dissemination. The presence and the number of involved lymph nodes remains the single best indicator of whether or not the cancer has become widely metastatic. To further confirm the data from the publically available datasets, we performed a comparative analysis of *DLC1* levels in primary tumors and associated nodal metastases. Using qRT-PCR analysis, no significant difference in *DLC1* expression was observed in lymph node metastases compared with their matched primary breast tumors (n = 23 pairs; *p* = 0.2474 using Wilcoxon test of simple one-way ANOVA- GraphPad Prism 7.03;**Fig 1D).** These results suggest that loss of *DLC1* expression may occur at the primary tumor stage rather than in metastatic lesions.

### Lack of *DLC1* expression is not due to mutations and methylation in primary breast tumors

**Fig 2:**
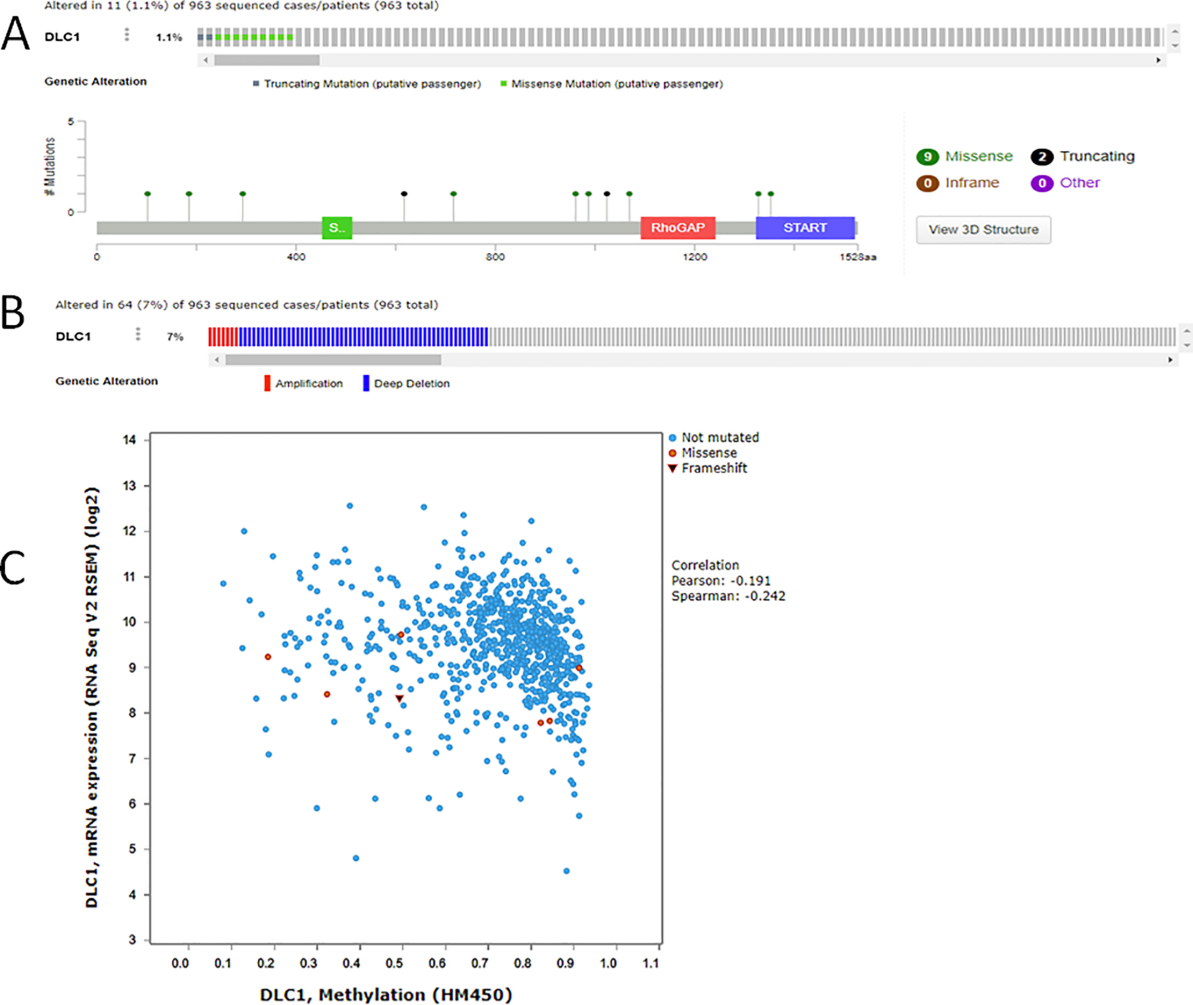
Genomic alterations in The Cancer Genome Atlas (TCGA) of breast invasive carcinoma (provisional). **A**. somatic mutations for DLC1, and **B**. Copy Number Alterations (CNA) Data were extracted from the TCGA of breast cancer dataset (BRCA) within the cBioPortal database. In this graphical summary, individual cases (patients) are represented as columns. **C.** The datasets used to analyze the methylation pattern at CpG islands using HM450 (Human Methylation 450 BeadChip) assay.

To determine the basis of low expression in primary breast tumors, we analyzed the genomic alterations in The Cancer Genome Atlas (TCGA) breast cancer dataset using the cBioPortal [20, 21]. Mutations within the coding region were found in ~1% of cases sequenced. These mutations were not located in the major structure domains associated with the protein function (**Fig 2A**). The copy number alterations (CNA) were identified in 7% of patients with majority being deep deletions (**Fig 2B**). We next analyzed 737 out of 1104 cases available with methylation data (HM450). There was poor correlation between RNA expression and methylation status and/or mutation status (Correlation Pearson: -0.147 and Spearman: -0.221) (**Fig 2C**). These analyses suggest that downregulation of *DLC1* in majority of breast tumors is not due to mutations or methylation.

### Functional impact of *DLC1* overexpression in ER+ breast cancer

**Fig 3:**
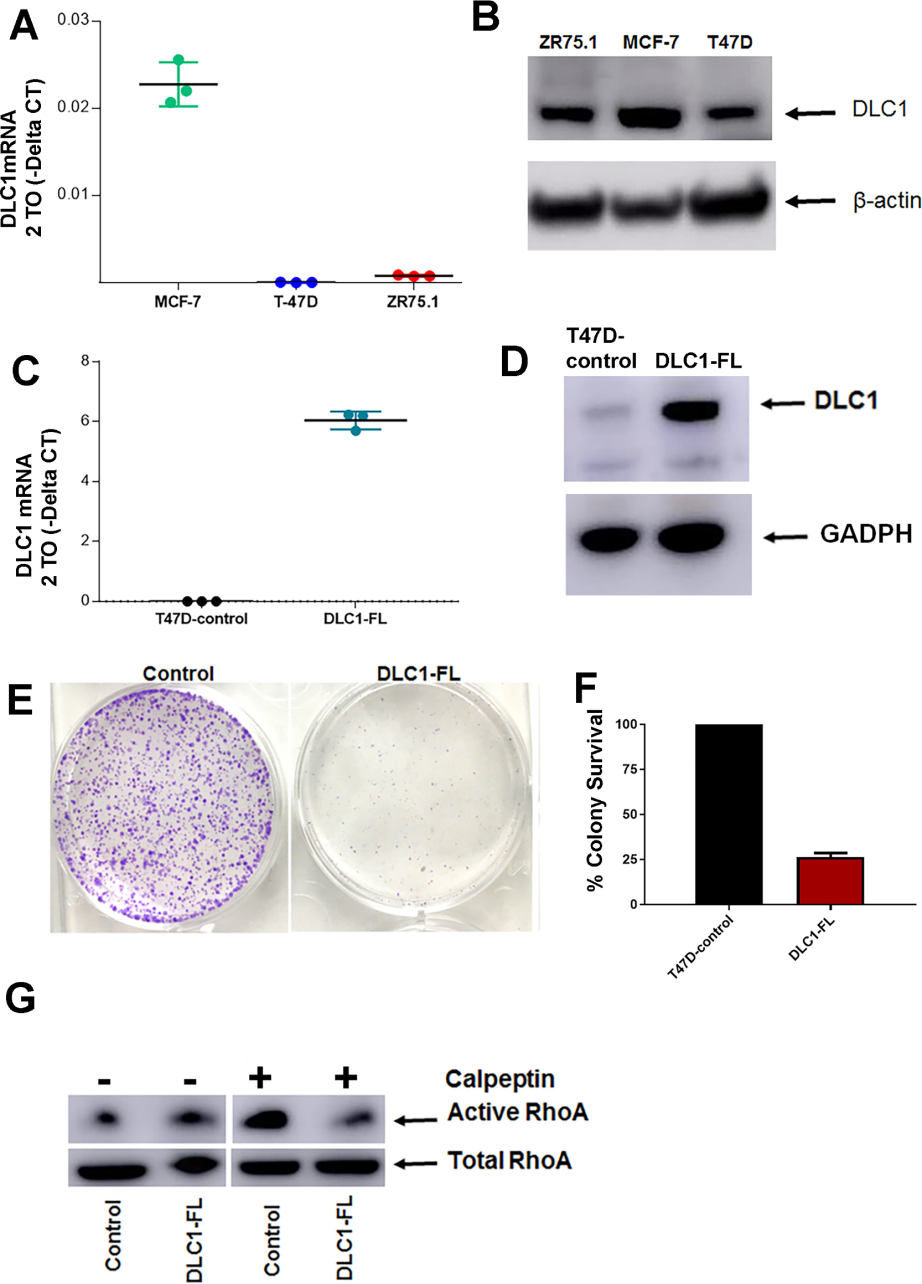
Overexpression of DLC1 decreases the colony formation significantly in ER+ breast cancer model. **A- B**. DLC1 expression in breast cancer cell lines, **A**. qRT-PCR, **B**. Western Blot. **C-D.** Confirmation of stable overexpression of DLC1-full length (DLC1-FL) expression compared to control vector (control) cells. **C**. qRT-PCR and **D**. Western blot. All qRT-PCR and Western blot assays are done in triplicates. **E-F**. Clonogenic assay documenting the quantitative decrease in colony formation in DLC1-FL cells (14 days). **G**. RhoA activation assay showing decreased RhoA activity in DLC1-FL cells. Calpeptin-activated RhoA activity is decreased in DLC1-FL compared to control cells. Total RhoA levels are similar in both cells. Representative data are shown from three separate experiments for clonogenic assay, western Blotting and RhoA activation assay.

We next measured the *DLC1* mRNA expression levels in ER+ breast cancer cells. Quantitative reverse transcription- PCR (qRT-PCR) analysis showed that ZR75-1 cells have very low levels of *DLC1*, while T-47D cells does not express *DLC1* at the detectable levels when compared with MCF-7 cells (both comparisons having adjusted *p* = 0.0001 using one-way ANOVA-multiple comparisons-GraphPad Prism 7.03; **Fig 3A**). Using Western blot, we also confirmed that MCF-7 has higher levels of DLC1 compared to T-47D and ZR75.1 cells (**Fig 3B**) To understand the functional relevance of *DLC1* in ER+ breast cancer, we established stable *DLC1* overexpression in T-47D (no endogenous expression of *DLC1*) cells using GenScript's GenEZ ORF cloning technology. The overexpression resulted in dramatic increase in *DLC1* mRNA levels in T-47D cells compared to the T-47D cells expressing control empty vector (*DLC1-FL* versus control) using qRT-PCR (**Fig 3C**). Overexpression of *DLC1* in *DLC1-FL* cells was also confirmed at the protein level (*p* = 0.0001; **Fig 3D**). Next, we have demonstrated that overexpression of *DLC1* exhibited significant quantitative reduction in colony formation (**Fig 3E and 3F**). Despite the effect of *DLC1* overexpression on cell growth, no changes were observed in cell cycle, and apoptosis in vitro (**S2 Fig**). To determine the alterations in invasiveness of the cells, we employed invasion assay in the presence or absence of serum in collagen 1-coated plates. This analysis showed a trend towards decreased invasiveness of the *DLC1-FL* cells (**S3 Fig**).

Next, we assessed the impact of *DLC1* overexpression on RhoA activation. Rho signaling is important for rearrangement of cancer cell cytoskeleton, which is crucial in cell proliferation and motility. As shown in **Fig 3G**, overexpression of DLC1 (*DLC1-FL*) resulted in decreased RhoA activation compared to control cells that lack DLC1, suggesting the Rho-inhibiting activity of DLC1 in this model.

### Proteomic and phosphopeptide analyses

**Fig 4:**
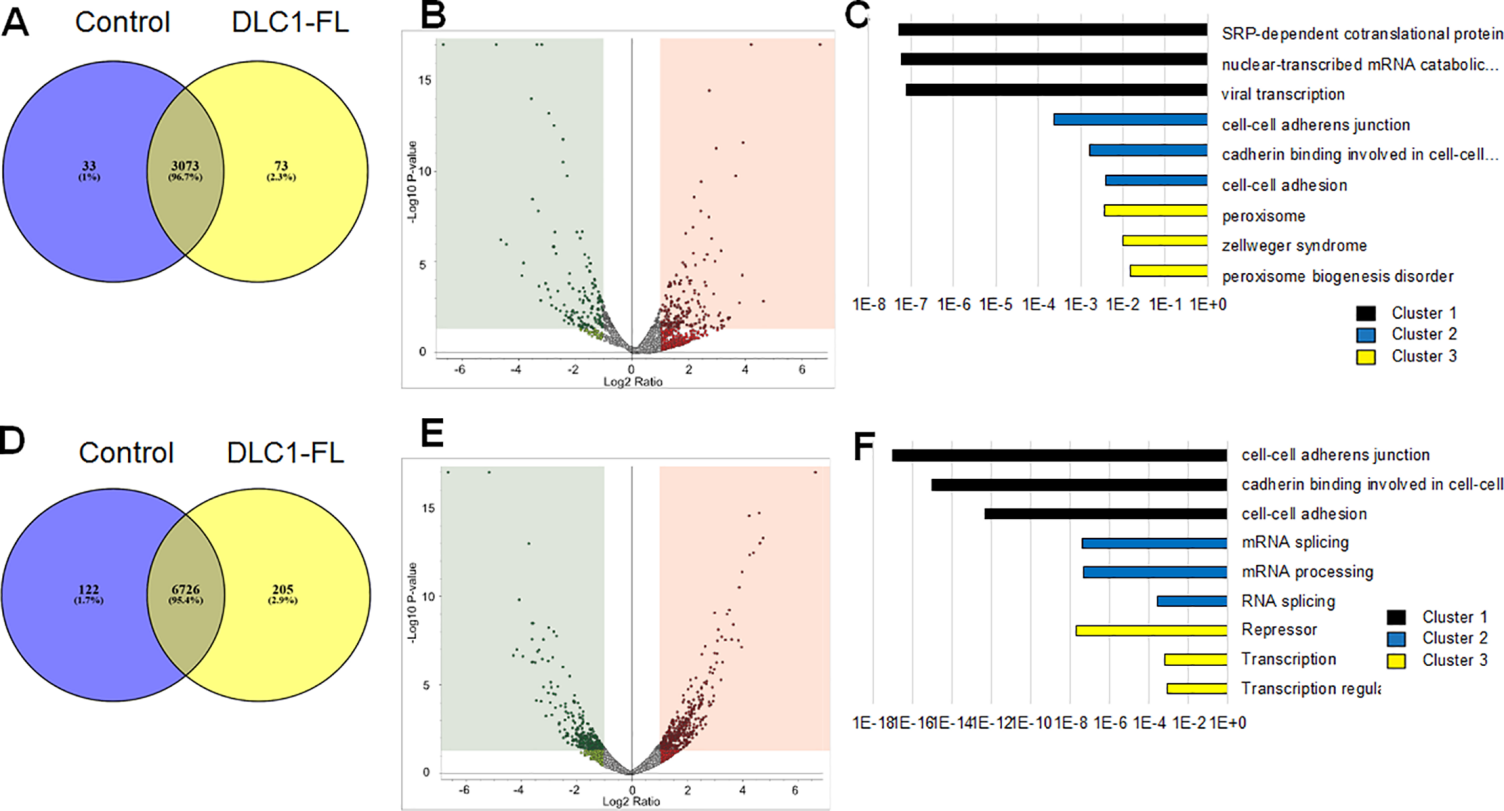
Label-free quantitative global proteomic and phosphoproteomic profiling of DLC1 overexpressing T-47D breast cancer cells. **A.** Venn diagram showing the identified global proteins in DLC1-FL and control cells. **B**. Volcano plots showing the differential protein expression (FDR-corrected *p*-value < 0.1) in DLC1-FL versus control cells. Red triangles represent differentially upregulated proteins, whereas green triangles show the differentially downregulated proteins. **C.** Pathway analysis clusters (DAVID) of the significantly altered protein expression in DLC1-FL versus control vector cells. **D** Venn diagram showing the identified phosphopeptides in DLC1-FL and control vector cells. **E**. Volcano plots showing the differential expressed phosphopeptides (FDR-corrected *p*-value < 0.1) in DLC1-FL versus control vector cells. Red triangles represent differentially upregulated phosphopeptides, whereas green triangles show the differentially downregulated phosphopeptides. **F**. Pathway analysis clusters (DAVID) of the significantly altered phosphopeptides in DLC1-FL versus control vector cells; DLC1-full-length and control vector T-47D cells were named as DLC1-FL and control, respectively.

DLC1 can regulate cellular signaling through protein-protein interactions. In particular, DLC1 can be regulated by phosphorylation [14]. In order to better understand the functionality of full-length DLC1 in ER+ breast cancer, we first performed proteomic analysis of control vector and DLC1-full-length (*DLC1-FL*) cells. The reproducibility of global proteome and phosphoproteome data is demonstrated in scatterplots for replicate samples in **S4** and **S5 Figs**, respectively. A total of 3,073 (96.7%) proteins were identified and quantified in both cell lines, while only 33 (1%) and 73 (2.3%) proteins were specifically identified and quantified in control vector cells and *DLC1-FL*, respectively (**Fig 4A**). More specifically, of the 397 differentially expressed proteins, 173 proteins were significantly depleted (FDR-corrected *p*-value < 0.1, log_2_ fold change < -1.0) in the *DLC1-FL* cells compared to control, whereas 224 proteins were significantly enriched (FDR-corrected *p*-value < 0.1, log_2_ fold change > 1.0) in *DLC1-FL* compared to control (**Fig 4B**; **S2–S4 Tables**). Using the DAVID Functional Annotation Clustering Tool, we identified the biological processes altered significantly in *DLC1-FL* overexpression (**Fig 4C; S5 Table**). The top three clusters consist of SRP-dependent co-translational protein targeting to membrane (cluster 1), cell-cell adhesion (cluster 2), and peroxisome signaling (cluster 3).

Further phosphoproteomic analysis identified 6,726 (95.4%) phosphopeptides that were identified and quantified in both cell types, while only 205 (2.9%) and 122 (1.7%) phosphopeptides were specifically identified and quantified in DLC1-FL and control vector cells, respectively (**Fig 4D**). Of the 1,301 phosphopeptides differentially quantified, 503 were significantly depleted (FDR-corrected *p*-value < 0.1, log_2_ fold change < -1.0) in *DLC1-FL* compared to control and 798 were significantly enriched (FDR-corrected *p*-value < 0.1, log_2_ fold change > 1.0) in *DLC1-FL* compared to control (**Fig 4E**; **S6–S8 Tables**). Of the top three clusters, cluster 1 consists of cell-cell adhesion signaling as expected (Benjamini corrected *p*<1E-12). Cluster 2 was specific to mRNA splicing and processing. Cluster 3 consisted of transcription regulation including repression (**Fig 4F**). Other important clusters have been listed in **S9 Table.**

**Fig 5:**
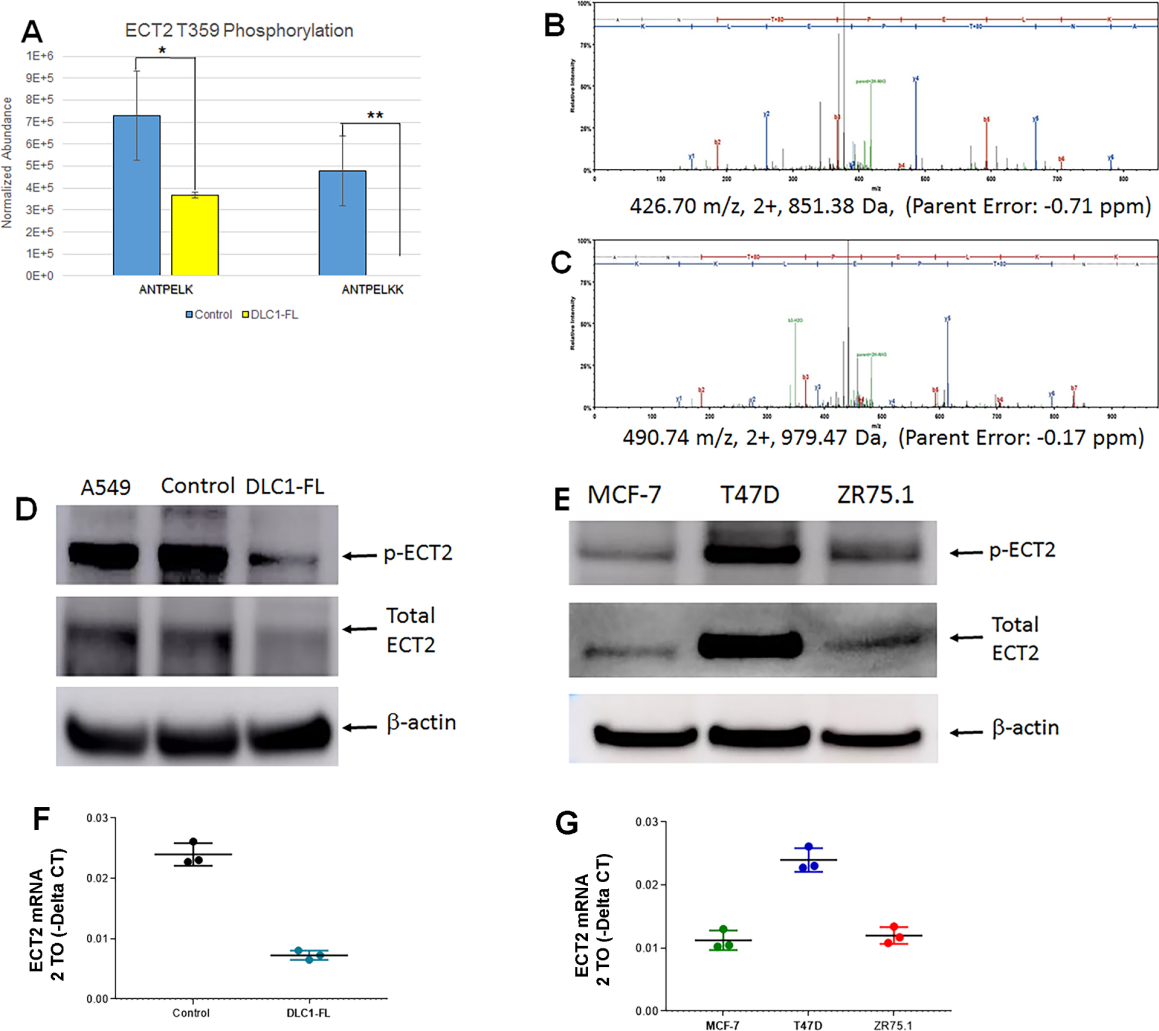
Decrease of ECT2 phosphorylation at T359 residue in DLC1 overexpressing cells. **A.** Significant decrease in two phosphopeptides (ANTPELK) and (ANTPELKK, with one missed cleavage) in DLC1-FL overexpressing cells compared to control vector cells (FDR-corrected *p*-value < 0.1). **B-C**. Phosphopeptide quantification by label-free LC-MS/MS. Representative mass spectra for each of the identified T359 phosphopeptides. **D**. Independent Western blot validation of ECT2 phosphorylation with phosphosite-specific and total protein antibodies in DLC1-FL and control vector cells (see details in “Materials and Methods”). A549 human lung carcinoma cell line was used as positive control for ECT2 phosphorylation and total protein levels. Antibody against β-actin was used as reference (loading control); p-ECT2 represents phospho ECT2 specific to T359 residue (Uniprot sequence identifier for isoform 2: Q9H8V3-2). **E**. ECT2 phosphorylation (T359) and total protein levels in ER+ breast cancer cell lines. **F**. ECT2 mRNA levels of control and DLC1-FL overexpressing cells using qRT-PCR. **G**. ECT2 mRNA levels of ER+ breast cancer cell lines using qRT-PCR assay.

Of the top phosphopeptides identified, we have focused on the epithelial cell transforming sequence 2 (*ECT2*) due to the availability of the antibody against the phosphopeptide. ECT2, a guanine nucleotide exchange factor for Rho family GTPases that catalyzes the exchange of GDP for GTP and activates the Rho GTPases [37]. *ECT2* has been reported to be overexpressed in a variety of human tumors including breast cancer [38–44]. In the METABRIC dataset of breast tumors from 1,992 patients, high *ECT2* expression was correlated significantly with poorer survival including all breast cancer subtypes [44]. *ECT2* function is regulated by phosphorylation [33, 45]. In our study, two phosphopeptides (ANTPELK) and (ANTPELKK, with one missed cleavage) of ECT2 were significantly reduced in *DLC1-FL* overexpressing cells compared to control cells (**Fig 5A**; FDR-corrected *p*-value < 0.1). A representative mass spectrum for each of the identified T359 phosphopeptides has been shown in **Fig 5B and 5C**. This phosphopeptide (T359) sequence is identical to the one previously reported as for T328 for a different isoform of ECT2 [33].

### Validation of ECT2 phosphorylation

Using Western blot analysis, we validated the decrease of phosphorylation at T359 as well as decrease of total protein levels of ECT2 in cells overexpressing DLC1-FL (**Fig 5D**). The ratio of the phospho-protein to the total protein showed decreased phospho-protein levels after normalization **(S6 Fig)**. Addition of ECT2 phosphospecific peptide as a competitor during antibody incubation completely abolished the bands thereby verifying the specificity of the bands (data not shown). Together, these data suggest that high DLC1 expression reduces the phosphorylation and protein levels of ECT2.

To determine whether our findings in T-47D model are cell-line specific or occur in other ER+ breast cancer cell lines, we evaluated ECT2 phosphorylation and protein levels in other ER+ breast cancer cell lines with various endogenous levels of DLC1 expression (**Fig 5E**). MCF-7 cells with higher DLC1 expression had the lower ECT2 phosphorylation and total ECT2 protein expression levels. ZR75.1 cells, as expected, also exhibited a lower level of both ECT2 phosphorylation and protein levels than T-47D cells. These data confirms that the inverse association of ECT2 and DLC1 is not cell-line specific and occur in other ER+ models. These findings are also verified at the mRNA levels (**Fig 5F and 5G).**

### ECT2 phosphorylation in the Clinical Proteomic Tumor Analysis Consortium (CPTAC) Data

We next compared data from a CPTAC breast cancer phosphoproteomics study [34] to data from this study. Unique phosphopeptide sequences, which were detected in the CPTAC study and were significantly changed in our study, are listed along with the matched Uniprot accession numbers and protein descriptions (**S10 Table**). Importantly, the ECT2 T359-containing phosphopeptide was detected in both basal and luminal patient-derived breast cancers.

### DLC1 overexpression decreases the expression of primary canonical ER target proteins independent of ER expression

To determine whether DLC1 overexpression alters the protein levels and the phosphopeptide levels of ER and endogenous ER targets, we assessed the protein levels of ER, trefoil factor 1/pS2, cyclin D1, and c-Myc, which are well-characterized ER-regulated genes. No peptides from ER, pS2, cyclin D1 or c-Myc were detected in either the global or the phospho-enriched samples in control or *DLC1-FL* cells. This is not surprising due to the untargeted approach that we utilized and the low levels of these proteins in the cells. Furthermore, progesterone receptor protein and phosphorylation levels were unchanged in *DLC1-FL* compared to control cells (FDR corrected p-values > 0.83)**(S7 Fig**).

**Fig 6:**
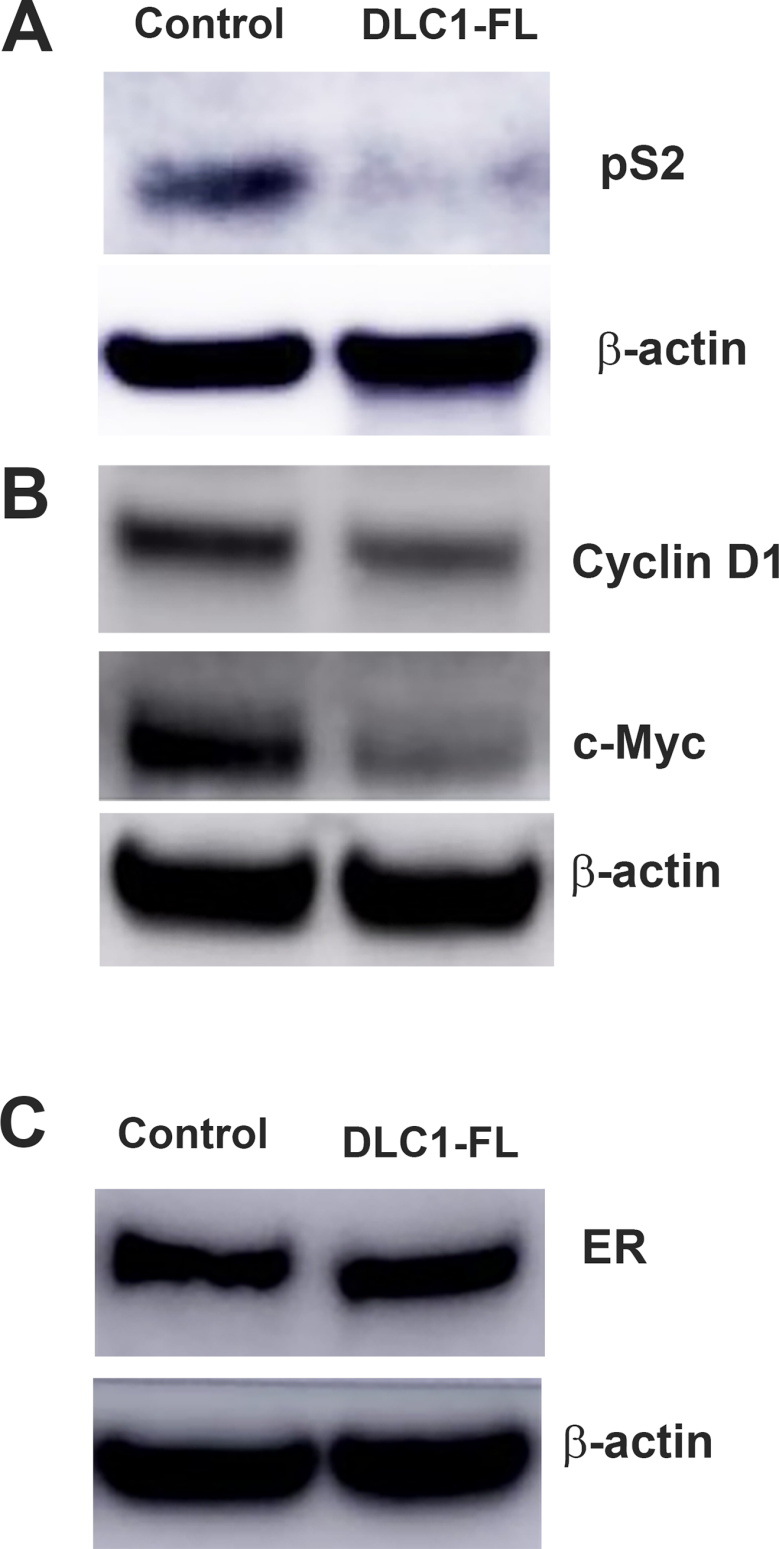
Overexpression of DLC1 decreases the expression of ER targets. Protein expression levels of **A.** pS2, **B.** cyclin D1 and c-Myc, and **C.** Estrogen Receptor (ER) in DLC1-FL versus control cell lines using Western blot analysis. β-actin is used as the loading control. The data are representative of three individual sets.

We further analyzed the protein levels expression of the above ER targets using Western blotting analysis. We demonstrated that *DLC1* overexpression decreased the protein expression of trefoil factor 1/pS2, cyclin D1, and c-Myc, although these were not detectable from proteomics data (**Fig 6**). However, the ER levels were not changed in both control and *DLC1-FL* cells. Phospho-ER levels were not at the detectable level in Western blotting (data not shown). These data suggests the effect of DLC1 in ER targets by mechanisms that are independent of ER.

## Discussion

In this study, we evaluated the expression and prognostic relevance of *DLC1* in breast cancer as well as its cellular interaction via phosphopeptide analysis in breast cancer. Our analyses revealed that high expression of *DLC1* correlated with longer overall survival (OS) in ER-positive, but not ER-negative patients. Of note, the association of low *DLC1* expression with poor outcome was prominent even in node-positive tumors.

Expression of *DLC1* is silenced in human gastric cancer [46], prostate cancer [7], multiple myeloma [47] and leukemia [48] cell lines. Our analysis of genomic alterations using TCGA revealed that downregulation or loss of *DLC1* is not due to copy number alterations (CNAs) or the mutations. This suggests that silencing of *DLC1* might be due to the epigenetic mechanisms. Hypermethylation of *DLC1* promoter has also been shown to be responsible for inactivating *DLC1* gene in various solid tumors [6, 9, 49] albeit in varying frequencies. Seng et al[6] reported that *DLC1* was methylated in 5 out of 14 (36%) primary breast carcinomas while Teramoto et al[49] found it to be is less frequent (10%). In concordance with the latter study, our analysis of TCGA breast dataset showed a methylation in 6% (44/737) of breast tumors. These findings suggest the contribution of other mechanisms such as transcriptional repression needs to be explored.

Dai et al recently reported that tumors with high *DLC1* and low *CDK6* expression were associated with good prognosis[50]. Although this study determined the relevance of *DLC1* in breast cancer within a subset population of CDK6 interaction, our study suggests the importance of *DLC1* in ER-positive breast cancer regardless of other factors. In their analysis of the TCGA dataset, they did not identify a strong prognostic impact for DLC1. The differences in the two studies could be due a number of factors including the limitations of treatment and follow-up information within the TCGA cohort.

In order to obtain additional insights into DLC1 function, we decided to study the protein-protein interactions. Phosphorylation is an important posttranslational modification that can determine the functional significance of the protein expression. Changes in the phosphoproteome can provide an insight into the regulation of the proteins. In our study, we applied a phosphopeptide enrichment analysis using full-length DLC1 in comparison with the control cells lacking DLC1 to identify altered phosphorylated peptides. This approach revealed the involvement of DLC1 in expected processes such as cell-cell adhesion as well as novel biological processes including mRNA processing. These data further opens new avenues to understand the DLC1 network in this cancer.

The differentially expressed phosphopeptides included ECT2, which has been documented to have multiple functions including cell cycle and ribosomal biogenesis [45, 51, 52]. We have identified the downregulation of the phosphopeptide corresponding to T359 of ECT2 in *DLC1-FL* cells validated using Western blotting. The specific sequence of this site (ANTPELK) has previously been shown to be phosphorylated by PKCι-Par6 at the T328 residue of ECT2 by the Justilien et al [33]. The phosphopeptide sequence for T328 is identical to the T359. The difference in the phosphosites could be due to an isoform difference. T359 corresponds to isoform 2 (Uniprot sequence identifier for isoform 2: Q9H8V3-2), whereas isoform 1 corresponds to T328. The comparison of the cell line with CPTAC confirmed ECT2 phosphopeptide in patient derived xenografts (PDXs) of breast cancers [34]. This is the first time, that ECT2 phosphorylation has been directly implicated in breast cancer. The importance of this phosphorylation site has been previously documented in lung cancer and proposed as a target for therapeutics [52].

In support our data, a recent study reported that several cancer types are associated with increased expression and activity of the ECT2 RhoGEF and decreased expression and activity of the DLC1 RhoGAP, leading to increased RhoA activity [53]. Using the TCGA and CPTAC databases, they examined the relationship between DLC1 and ECT2 and supported our findings in this study. Indeed, they also stated that the combination of high ECT2 expression and low DLC1 expression level is more common in poorly differentiated tumors than in well differentiated ones. Together, both studies suggest that ECT2 and DLC1 frequently act together, but in opposite directions, in cancer to increase RhoA activity. Interestingly, our data also suggests the effect of DLC1 on ER targets independent of ER expression.

In conclusion, our study suggests a role of *DLC1* in prognosis of ER-positive breast cancer. DLC1 decreased the expression of primary canonical ER target proteins independent of ER expression. Furthermore, our phosphoproteomic analysis, for the first time, demonstrated an inverse correlation with high DLC1 expression and lower phosphorylation of ECT2 in breast cancer. Further analyses are necessary to determine the potential targeting of ECT2 in breast cancers with low DLC1 expression.

## Supporting information

S1 Table Patient characteristics and standard biomarker expression status of the 23-cases used in the study. (XLSX)

S2 Table Global protein level MS/MS results from Proteome Discoverer 2.2- DLC1-FL overexpressing versus control cells. (XLSX)

S3 Table Global peptide level MS/MS results from Proteome Discoverer 2.2- DLC1-FL overexpressing versus Control Cells. (XLSX)

S4 Table Global PSM level MS/MS results from Proteome Discoverer 2.2-DLC1-FL overexpressing versus Control Cells. (XLSX)

S5 Table **DAVID Functional Annotation Clusters in DLC1-FL overexpressing versus control cells-global protein level MS/MS results from Proteome Discoverer 2.2, A. Upregulated. B. Downregulated proteins**.
(XLSX)

S6 Table Phosphoenriched protein level MS/MS results from Proteome Discoverer 2.2-DLC1-FL overexpressing versus control cells. (XLSX)

S7 Table Phosphoenriched peptide level MS/MS results from Proteome Discoverer 2.2-DLC1-FL Overexpressing versus Control Cells. (XLSX)

S8 Table Phosphoenriched PSM level MS/MS results from Proteome Discoverer 2.2-DLC1-FL overexpressing versus control cells. (XLSX)

S9 Table DAVID functional annotation clusters upregulated in DLC1-FL overexpressing versus control cells- Phosphoenriched protein level MS/MS results from Proteome Discoverer 2.2. A. Upregulated phosphoenriched proteins. **B**. Downregulated phosphoenriched proteins.
(XLSX)

S10 Table Phosphopeptide sequences shared between Mertins et al. 2014 [34] study and this study's significantly changed sequences. (XLSX)

S1 Fig Workflow for label-free quantitative global proteomic and phosphoproteomic analyses. (TIF)

S2 Fig Functional assays of DLC1-FL and control cells. (A), Cell cycle, (B) Apoptosis. Representative assays from at least three separate experiments for each cell line are shown.
(TIF)

S3 Fig Invasion assay of DLC1-FL and control cells. As a chemoattractant, 20% Fetal Bovine Serum (FBS) is used. Representative assays from at least three separate experiments for each cell line are shown.
(TIF)

S4 Fig Correlation plots of the normalized abundances (label-free quantitation; area under the curve) for proteins in the global analysis. (Top row) Comparisons for control replicates and (bottom row) DLC1-FL (full-length) replicates. Pearson correlation coefficients (R^2^) are listed in the top left corner for each comparison.
(AI) Click here for additional data file.

S5 Fig Correlation plots of the normalized abundances (label-free quantitation; area under the curve) for proteins in the phosphoenriched analysis. (Top row) Comparisons for control replicates and (bottom row) DLC1-FL (full-length) replicates. Pearson correlation coefficients (R^2^) are listed in the top left corner for each comparison.
(AI) Click here for additional data file.

S6 Fig The ratio of the phospho- protein to the total protein of ECT2 in DLC1-FL versus T47D-control cells. (PPTX)

S7 Fig Progesterone receptor protein and phosphorylation levels are unchanged in DLC1-FL. **A**. Normalized abundances for progesterone receptor in control and DLC1-FL cells. FDR corrected p-value = 0.88. **B.** Normalized abundances for phosphorylation of progesterone receptor in control and DLC1-FL cells. Ambiguous localization on a phosphopeptide is denoted with several residues listed. S162 phosphorylation was detected on a peptide with and without methionine oxidation. All FDR corrected p-values > 0.83.
(PPTX)

S1 Appendix qRT-PCR assays for T47D-DLC1 colony screening. (PPTX)
