# Supplementary figures and images for: Quantitative phosphoproteomic analysis identifies novel functional pathways of tumor suppressor DLC1 in estrogen receptor positive breast cancer
Source: PLoS One. 2018 Oct 2;13(10):e0204658. doi: 10.1371/journal.pone.0204658 (PMC6168143; doi:10.1371/journal.pone.0204658)

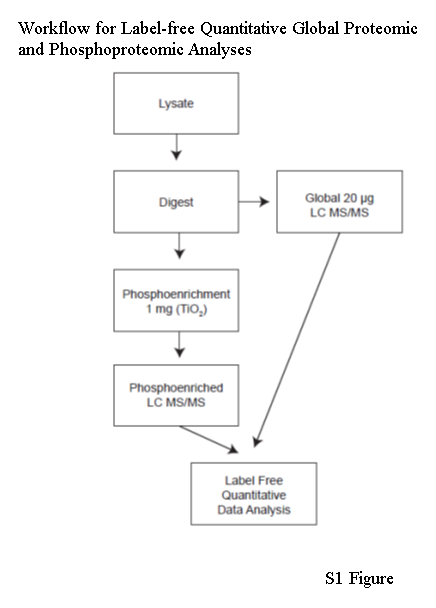

Supplement: S1 Fig — (TIF) [file pone.0204658.s011.tif]

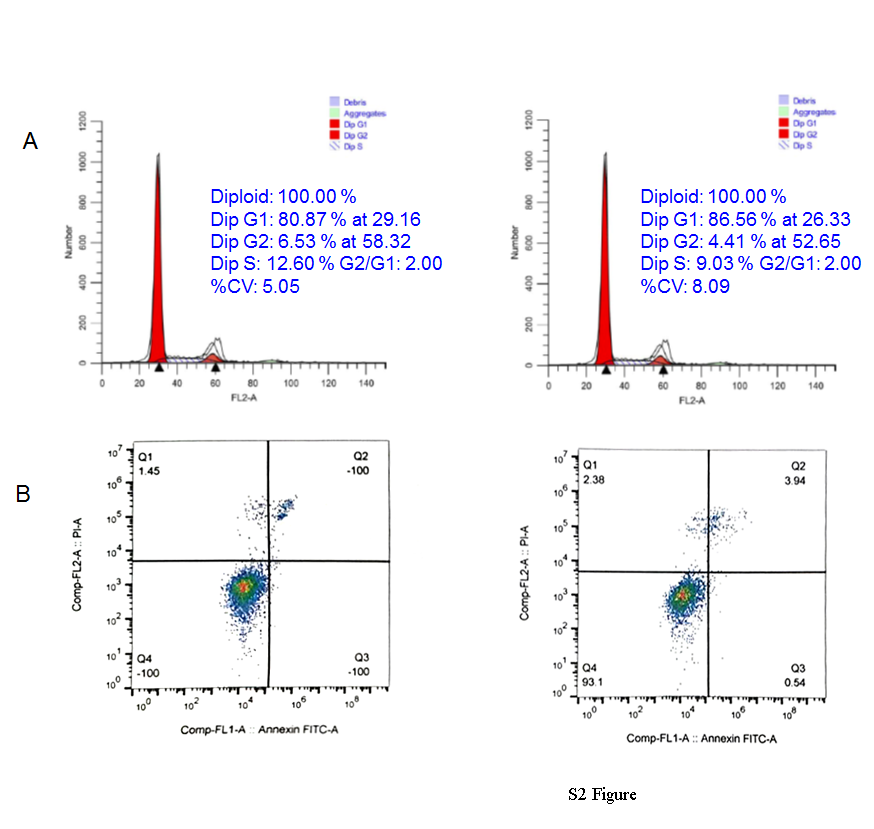

Supplement: S2 Fig — (A), Cell cycle, (B) Apoptosis. Representative assays from at least three separate experiments for each cell line are shown. (TIF) [file pone.0204658.s012.tif]

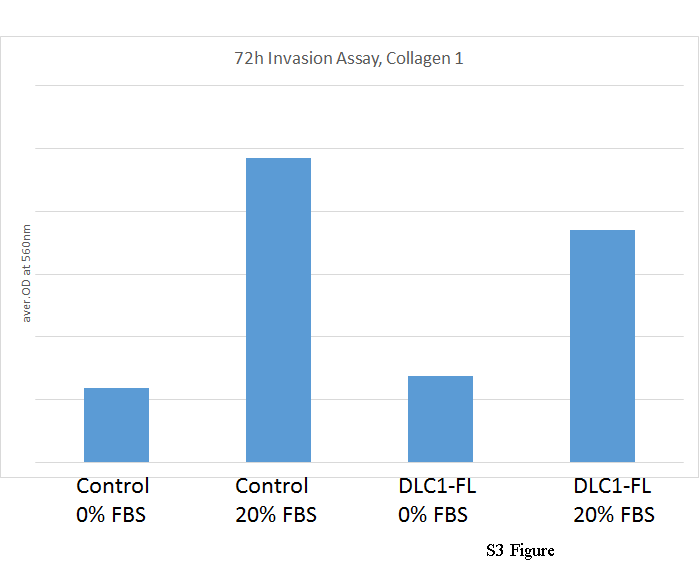

Supplement: S3 Fig — As a chemoattractant, 20% Fetal Bovine Serum (FBS) is used. Representative assays from at least three separate experiments for each cell line are shown. (TIF) [file pone.0204658.s013.tif]

## Slide 1
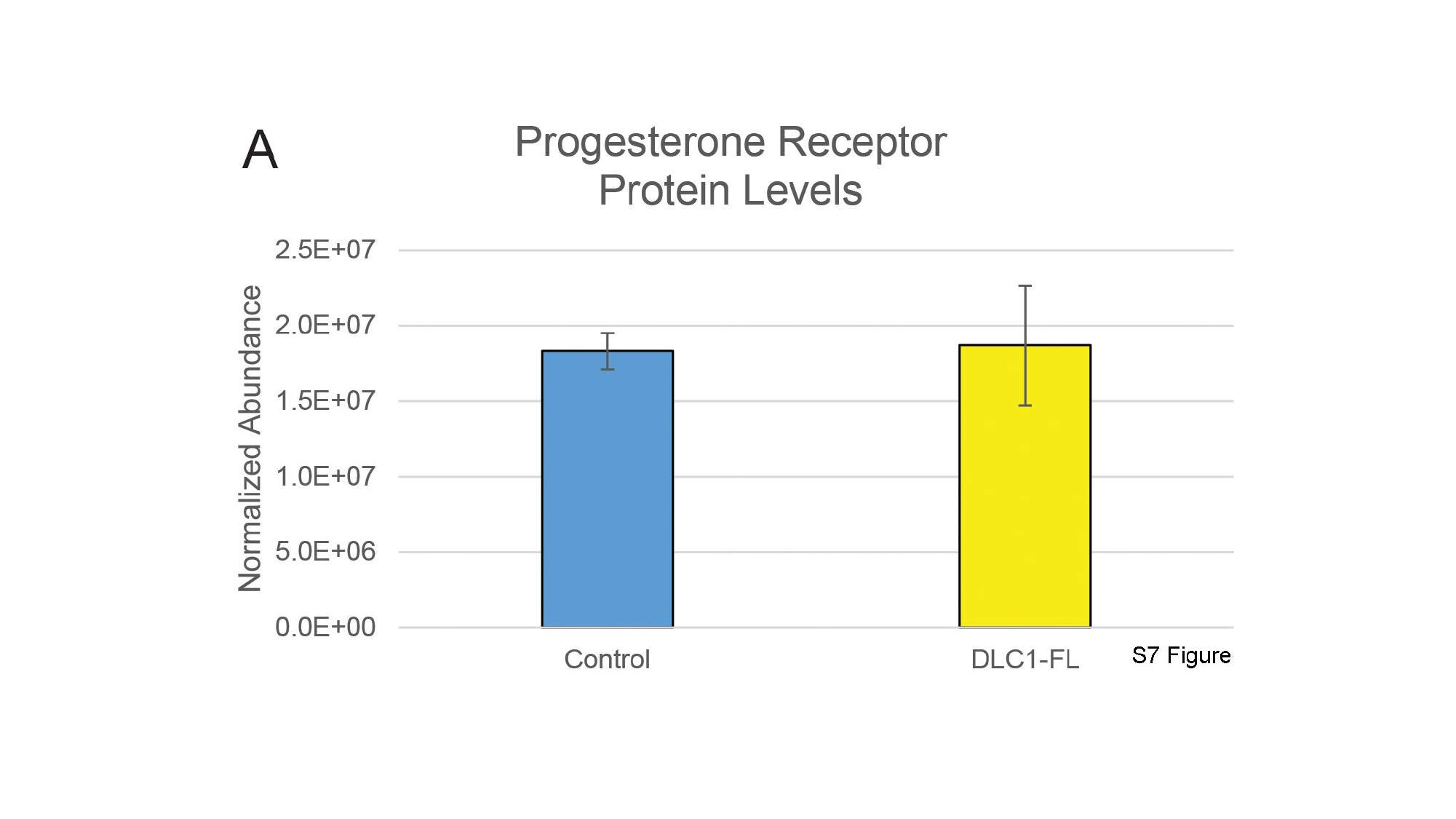

## Slide 2
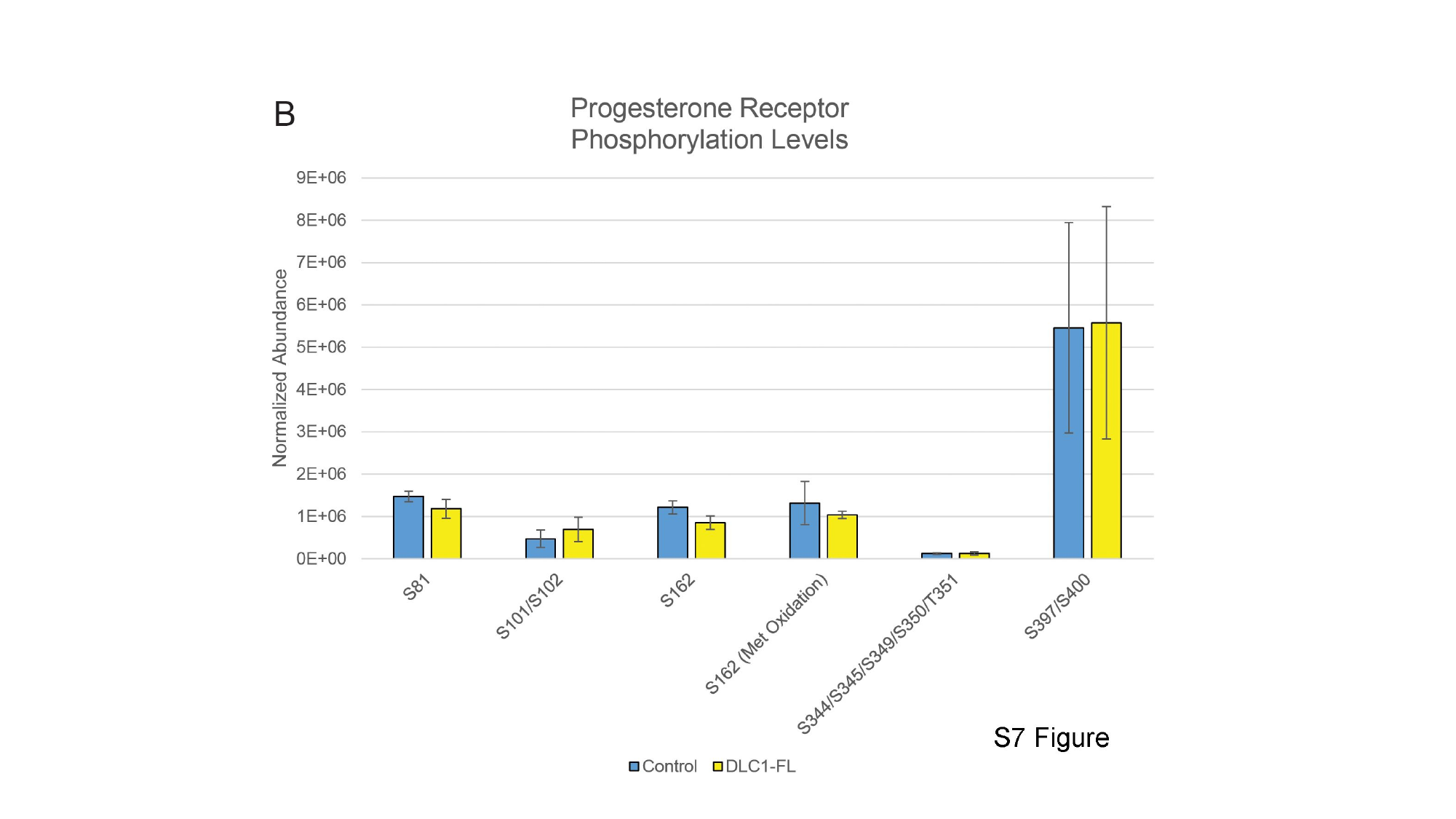

Supplement: S7 Fig — A. Normalized abundances for progesterone receptor in control and DLC1-FL cells. FDR corrected p-value = 0.88. B. Normalized abundances for phosphorylation of progesterone receptor in control and DLC1-FL cells. Ambiguous localization on a phosphopeptide is denoted with several residues listed. S162 phosphorylation was detected on a peptide with and without methionine oxidation. All FDR corrected p-values > 0.83. (PPTX) [file pone.0204658.s017.pptx]
