# Supplementary material for: Quantitative phosphoproteomic analysis identifies novel functional pathways of tumor suppressor DLC1 in estrogen receptor positive breast cancer
Source: PLoS One. 2018 Oct 2;13(10):e0204658. doi: 10.1371/journal.pone.0204658 (PMC6168143; doi:10.1371/journal.pone.0204658)
Supplement: S6 Fig — (PPTX) [file pone.0204658.s016.pptx]

## Slide 1
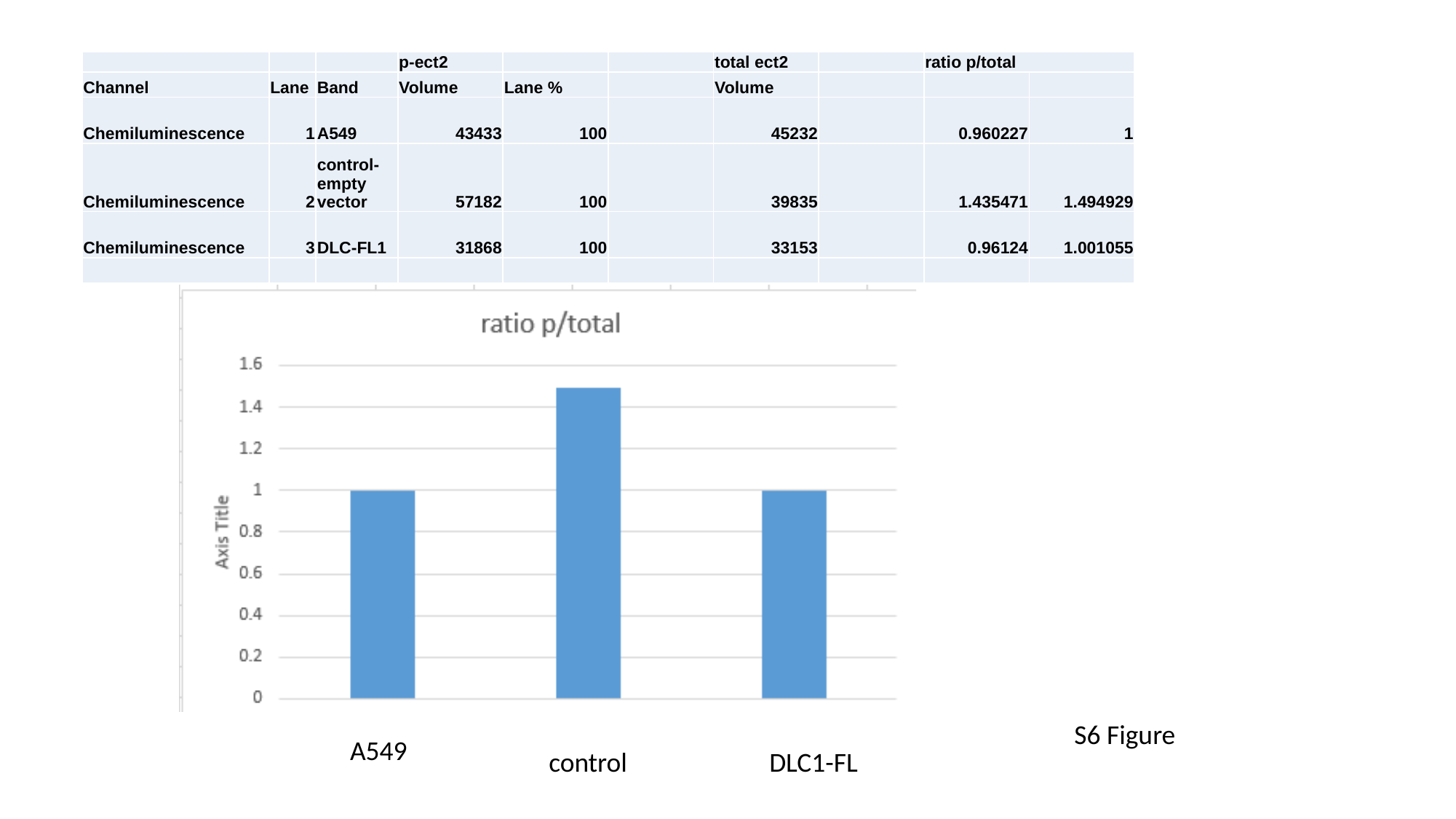

| | | | p-ect2 | | | total ect2 | | ratio p/total | |
| --- | --- | --- | --- | --- | --- | --- | --- | --- | --- |
| Channel | Lane | Band | Volume | Lane % | | Volume | | | |
| Chemiluminescence | 1 | A549 | 43433 | 100 | | 45232 | | 0.960227 | 1 |
| Chemiluminescence | 2 | control-empty vector | 57182 | 100 | | 39835 | | 1.435471 | 1.494929 |
| Chemiluminescence | 3 | DLC-FL1 | 31868 | 100 | | 33153 | | 0.96124 | 1.001055 |
| | | | | | | | | | |
S6 Figure
A549
control
DLC1-FL
