# Supplementary material for: Quantitative phosphoproteomic analysis identifies novel functional pathways of tumor suppressor DLC1 in estrogen receptor positive breast cancer
Source: PLoS One. 2018 Oct 2;13(10):e0204658. doi: 10.1371/journal.pone.0204658 (PMC6168143; doi:10.1371/journal.pone.0204658)
Supplement: S1 Appendix — (PPTX) [file pone.0204658.s018.pptx]

## Slide 1
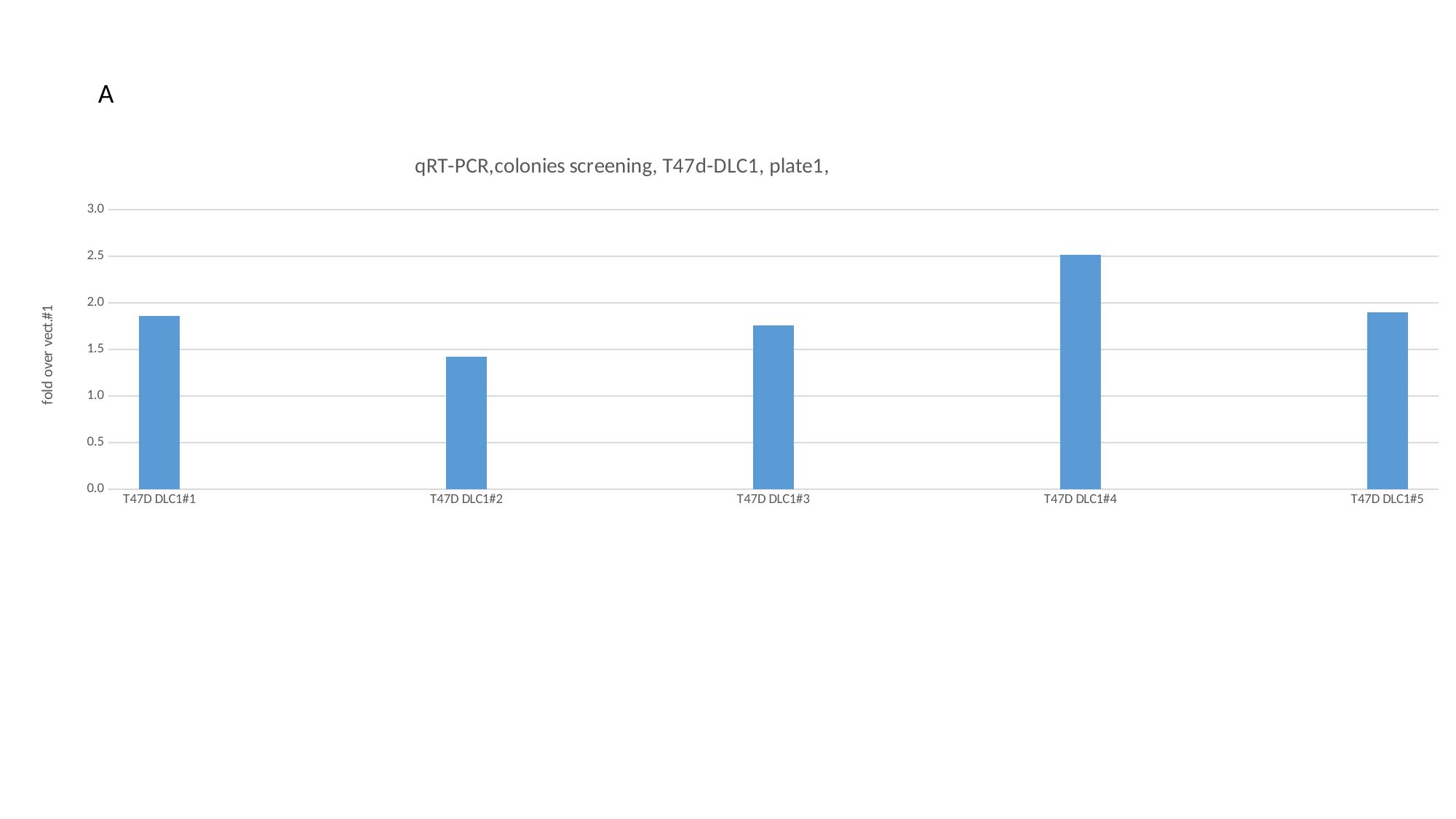

A
### Chart: qRT-PCR,colonies screening, T47d-DLC1, plate1,
| Category | |
|---|---|
| T47D DLC1#1 | 1.8601264096096133 |
| | None |
| | None |
| T47D DLC1#2 | 1.4185581913775276 |
| | None |
| | None |
| T47D DLC1#3 | 1.7597302975995086 |
| | None |
| | None |
| T47D DLC1#4 | 2.5107992214232646 |
| | None |
| | None |
| T47D DLC1#5 | 1.8983223577945045 |

## Slide 2
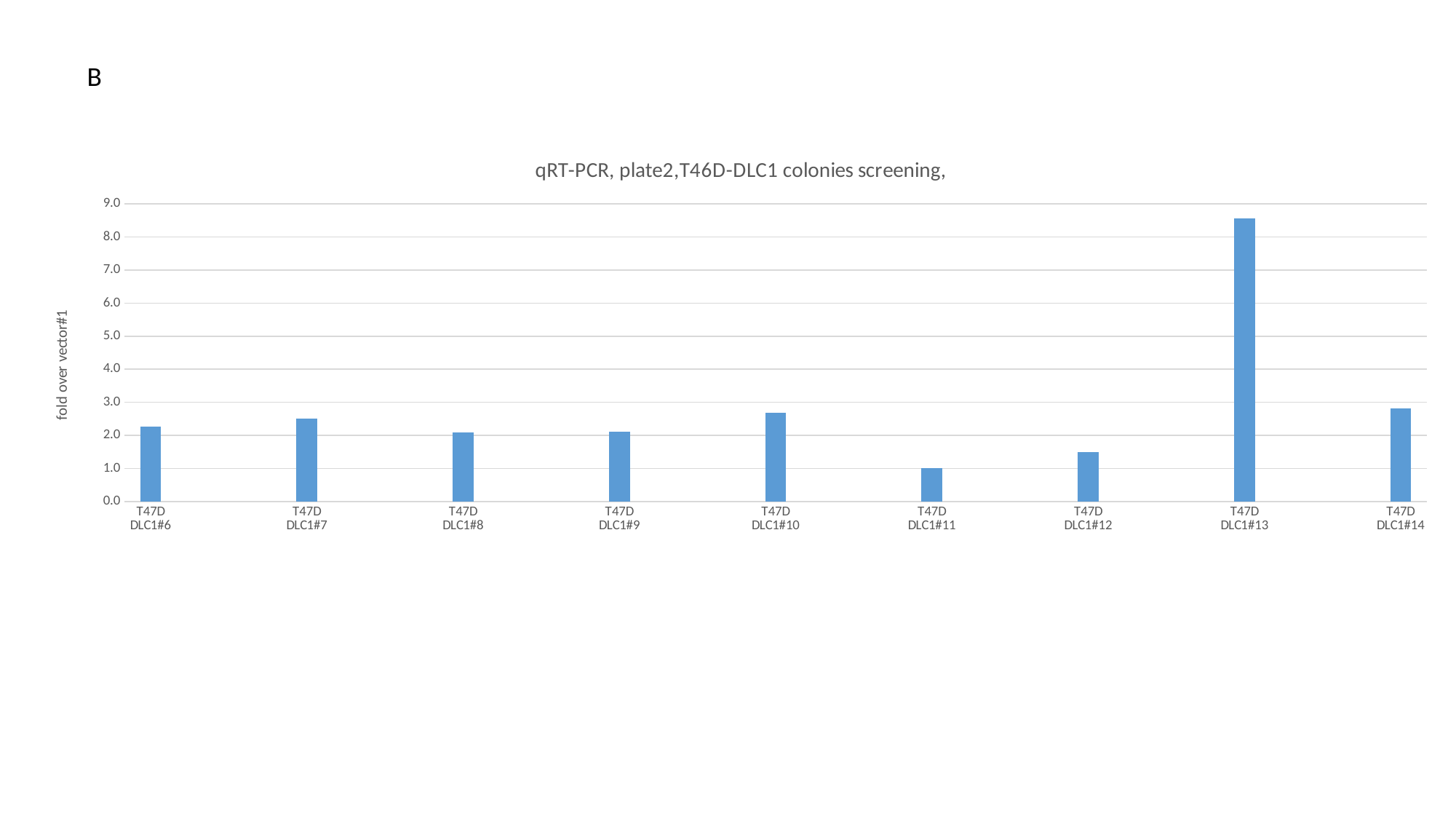

B
### Chart: qRT-PCR, plate2,T46D-DLC1 colonies screening,
| Category | |
|---|---|
| T47D DLC1#6 | 2.272874459203742 |
| | None |
| | None |
| T47D DLC1#7 | 2.5035044155953132 |
| | None |
| | None |
| T47D DLC1#8 | 2.093749239834194 |
| | None |
| | None |
| T47D DLC1#9 | 2.122608547617717 |
| | None |
| | None |
| T47D DLC1#10 | 2.6752023131911207 |
| | None |
| | None |
| T47D DLC1#11 | 1.0056992932597737 |
| | None |
| | None |
| T47D DLC1#12 | 1.4899896748754413 |
| | None |
| | None |
| T47D DLC1#13 | 8.558039473516377 |
| | None |
| | None |
| T47D DLC1#14 | 2.82249103771294 |

## Slide 3
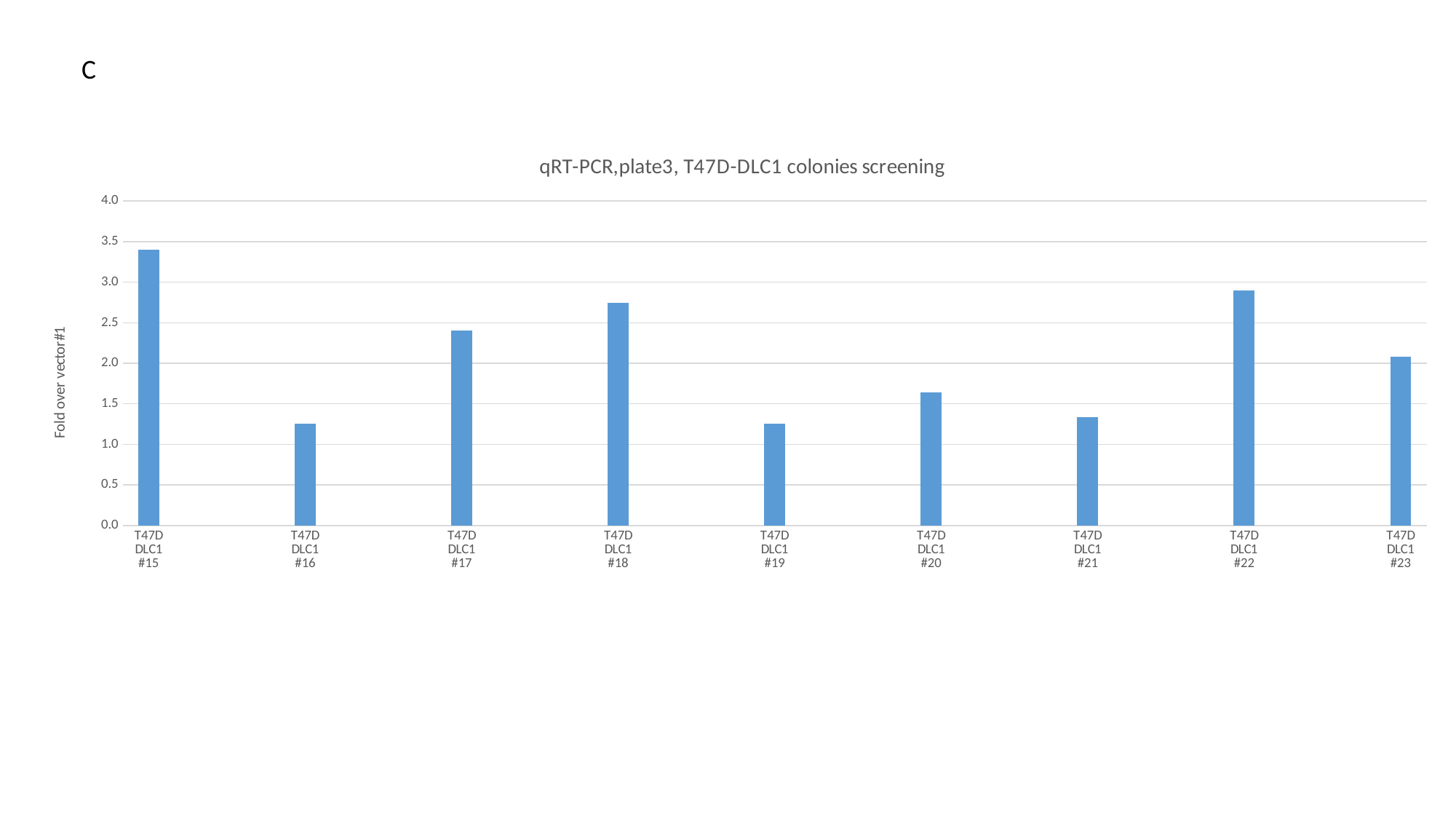

C
### Chart: qRT-PCR,plate3, T47D-DLC1 colonies screening
| Category | |
|---|---|
| T47D DLC1 #15 | 3.402498832428096 |
| | None |
| | None |
| T47D DLC1 #16 | 1.2510036510408389 |
| | None |
| | None |
| T47D DLC1 #17 | 2.4061106677285187 |
| | None |
| | None |
| T47D DLC1 #18 | 2.749037651828059 |
| | None |
| | None |
| T47D DLC1 #19 | 1.256263993616516 |
| | None |
| | None |
| T47D DLC1 #20 | 1.6403784167483004 |
| | None |
| | None |
| T47D DLC1 #21 | 1.3315900542230046 |
| | None |
| | None |
| T47D DLC1 #22 | 2.8958703229597997 |
| | None |
| | None |
| T47D DLC1 #23 | 2.0773581966594916 |
